# Supplementary material for: Overexpression of a Fragaria × ananassa AP2/ERF Transcription Factor Gene (FaTINY2) Increases Cold and Salt Tolerance in Arabidopsis thaliana
Source: Int J Mol Sci. 2025 Feb 27;26(5):2109. doi: 10.3390/ijms26052109 (PMC11900429; doi:10.3390/ijms26052109)
Supplement: Supplementary file 1 [file ijms-26-02109-s001.zip › Supplementary figure S1.pdf]

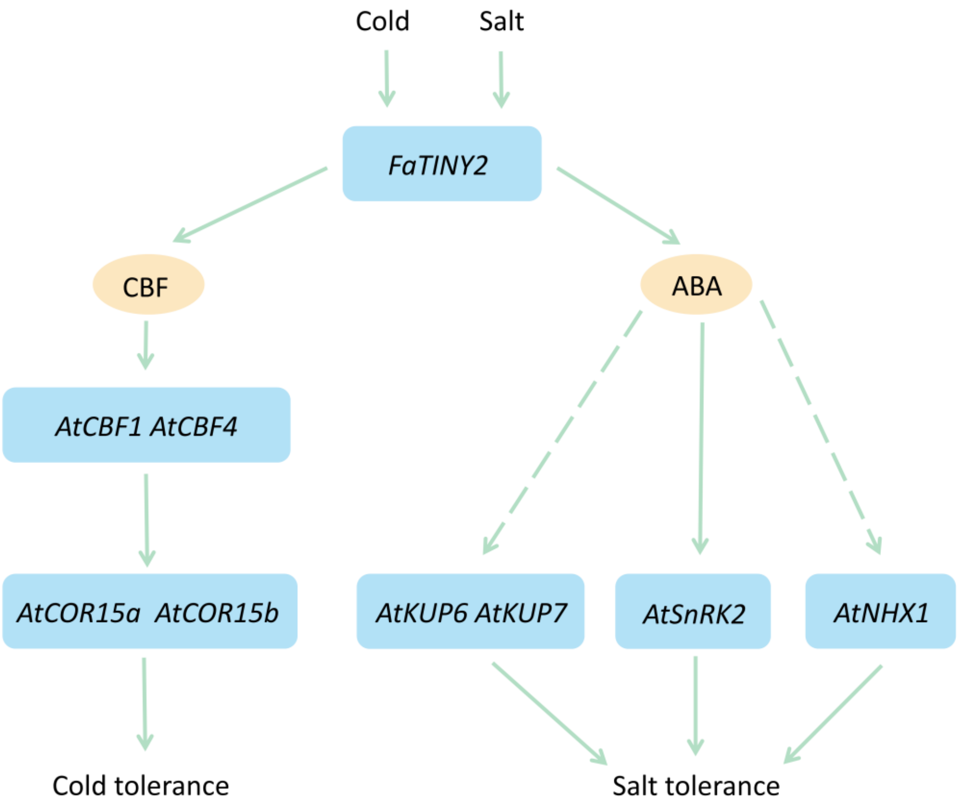

Figure S1. Working model of *FaTINY2* involved in regulation of plant cold stress and salt stress. When subjected to cold stress, *FaTINY2* is involved in the CBF pathway, binds to the *AtCBF1* and *AtCBF4* promoters, and stimulates the activation of subsequent genes *AtCOR15a* and *AtCOR15b*, thereby enhancing the cold tolerance of plants. *FaTINY2* plays a role when subjected to salt stress within the ABA pathway, enhancing the expression of *AtSnRK2*. The expression of *AtNHX1* regulates Na^+ transportation, and the expression of *AtKUP6* and *AtKUP7* regulates K^+ transport, indirectly engaging in the ABA pathway and boosting plant salt resistance.
