# Supplementary material for: Overexpression of a Fragaria × ananassa AP2/ERF Transcription Factor Gene (FaTINY2) Increases Cold and Salt Tolerance in Arabidopsis thaliana
Source: Int J Mol Sci. 2025 Feb 27;26(5):2109. doi: 10.3390/ijms26052109 (PMC11900429; doi:10.3390/ijms26052109)
Supplement: Supplementary file 1 [file ijms-26-02109-s001.zip › Supplementary table S1.pdf]

Supplementary Materials

Table S1. List of primers used in this study

Primer Name	PrimerSequence (5′ →3′)	Purpose
FaTINY2-F	ATGAGTTGTACTACTGAAACCCAGT	full-length cDNA of *FaTINY2*
FaTINY2-R	TTAATAATCCCATAACAAACCCT	full-length cDNA of *FaTINY2*
FaTINY2-slF	GAGCTCGGTACCCGGGGATCCATGAGTTGTACTACTGAAACCCAGT	For subcellular localization
FaTINY2-slR	GCTCACCATGTCGACTCTAGAATAATCCCATAACAAACCCT	For subcellular localization
FaTINY2-qF	TGCAACCCAAAAACAACGACA	qPCR
FaTINY2-qR	CGAGCCAAATGCGTGACTTC	qPCR
FaActin-qF	GGGCCAGAAAGATGCTTATGTCGG	qPCR
FaActin-qR	GGGCAACACGAAGCTCATTGTAGAAG	qPCR
AtActin-qF	GGATCTGTACGGTAACATTGTGC	qPCR
AtActin-qF	CTGCTGGAATGTGCTGAGG	qPCR
AtCBF1-qF	CGAGTGGAACATCGTGGGAT	qPCR
AtCBF1-qR	AACGGATATGTGGGGAGCAC	qPCR
AtCBF4-qF	GTACGGAACTCCGGACCAAT	qPCR
AtCBF4-qR	ACTTTGACGGAGTGGGTGAC	qPCR
AtCOR15a-qF	TCGCTTTCTCACCATCTGCT	qPCR
AtCOR15a-qR	CTGTTGACTACTTTTGGATATGTGT	qPCR
AtCOR15b-qF	GGCCTCCTTCGTCTTATCCG	qPCR
AtCOR15b-qR	TGGCCTTGTTGAGGATTGAGT	qPCR
AtNHX1-qF	GCACAGTGGTAAGAAATCTCTGG	qPCR
AtNHX1-qR	GTGGTTCCCTGAAGGCTCAA	qPCR
AtKUP6-qF	TGATGATGACTTACCTTGGCGT	qPCR
AtKUP6-qR	TCAACTCTTCACTTTCCAGTGGT	qPCR
AtSnRK2-qF	AACACCCAATCAGCTAACGA	qPCR
AtSnRK2-qR	GCTCTCCATGAGCTGGTGAT	qPCR
AtKUP7-qF	ATGTCCCGGTTCCAGTTGTA	qPCR
AtKUP7-qR	GGAAAAACAGACCAACCTGGC	qPCR
