# Supplementary figures and images for: Overexpression of a Fragaria × ananassa AP2/ERF Transcription Factor Gene (FaTINY2) Increases Cold and Salt Tolerance in Arabidopsis thaliana
Source: Int J Mol Sci. 2025 Feb 27;26(5):2109. doi: 10.3390/ijms26052109 (PMC11900429; doi:10.3390/ijms26052109)

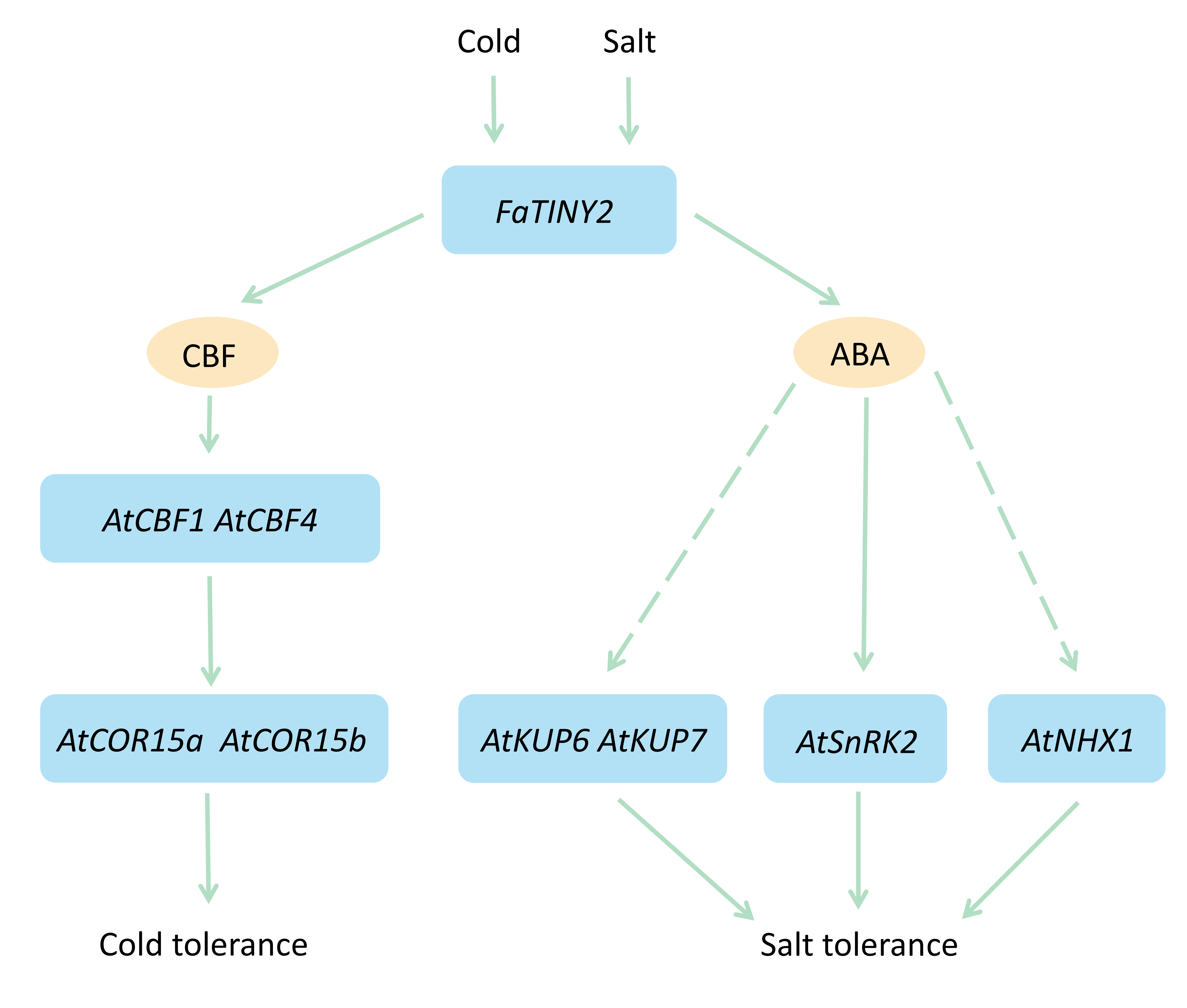

Supplement: Supplementary file 1 [file ijms-26-02109-s001.zip › Supplementary figure S1.TIF]
